# Explainable Deep Learning for Augmentation of Small RNA Expression Profiles

**DOI:** 10.1089/cmb.2019.0320

**Published:** 2020-02-06

**Authors:** Jelena Fiosina, Maksims Fiosins, Stefan Bonn

**Affiliations:** ^1^ Clausthal University of Technology, Institute of Informatics, Clausthal-Zellerfeld, Germany.; ^2^ German Center for Neurodegenerative Diseases, Tübingen, Germany.; ^3^ Institute for Medical Systems Biology, University Medical Center Hamburg-Eppendorf, Hamburg, Germany.; ^4^ Genevention GmbH, Göttingen, Germany.

**Keywords:** augmentation, classification, deep learning, explainable artificial intelligence, ontology, random forestsmall RNA expression

## Abstract

**The lack of well-structured metadata annotations complicates the reusability and interpretation of the growing amount of publicly available RNA expression data. The machine learning-based prediction of metadata (data augmentation) can considerably improve the quality of expression data annotation. In this study, we systematically benchmark deep learning (DL) and random forest (RF)-based metadata augmentation of tissue, age, and sex using small RNA (sRNA) expression profiles. We use 4243 annotated sRNA-Seq samples from the sRNA expression atlas database to train and test the augmentation performance. In general, the DL machine learner outperforms the RF method in almost all tested cases. The average cross-validated prediction accuracy of the DL algorithm for tissues is 96.5%, for sex is 77%, and for age is 77.2%. The average tissue prediction accuracy for a completely new data set is 83.1% (DL) and 80.8% (RF). To understand which sRNAs influence DL predictions, we employ backpropagation-based feature importance scores using the DeepLIFT method, which enable us to obtain information on biological relevance of sRNAs.**

## 1. Introduction

Data annotations (tissue, age, sex, etc.) are crucial for the reuse of data. A detailed description of the biological conditions in which data have been obtained is required to extract new information from the obtained data. The data should be findable, accessible, interoperable, and reusable, which ultimately facilitates knowledge discovery (Wilkinson et al., 2016). Annotations are an essential part of semantic data integration systems and allow for a deeper analysis of the data (Madan et al., 2018). So far, metadata are often not stored together with the expression data and the available metadata are often not normalized, and are unstructured and incomplete. The widely used GEO repository (Gene Expression Omnibus [GEO]; https://www.ncbi.nlm.nih.gov/geo), for example, stores annotations as a number of free text description fields. This leads to missing and/or inaccurate annotations and requires revisions and manual corrections by an expert (Hadley et al., 2017).

In this study, we aim to predict the metadata based on deep-sequenced small RNAs' (sRNAs') expression profiles by formulating this prediction as a classification problem. sRNAs are short (<200 nt), usually noncoding RNA molecules with many crucial biological functions (Storz, 2002). The basic rationale for this approach is that data with similar sRNA expression levels should have similar metadata. Based on this assumption, we hypothesize that sRNA expression profiles contain enough information to predict the sRNA tissue, age, and sex accurately. We believe that deep learning (DL)-based algorithms might outperform more conventional random forest (RF)-based machine learners (MLs) in sRNA metadata prediction, if enough training data are available. We also hypothesize that backpropagation-based feature importance scores may help to biologically rationalize the classification process of DL.

To distinguish between biological conditions, different ML methods were applied. In Guo et al. (2017) and Hadley et al. (2017), the sex in different microRNA (miRNA) tissue samples was defined using differential expression (DE) analysis. In Hadley et al. (2017), the authors used DE analysis and analysis of variance to detect the sex differences in several tissues in miRNAs. In Ellis et al. (2018), the age, sex, and tissue were predicted from mRNA sequencing (mRNA-Seq) expression data using a regression-based approach. massiR (Buckberry et al., 2014) is a method for sex prediction based on gene expression microarrays using clustering. Many studies use an RF method for the classification of expression data, particularly in disease diagnostics (Statnikov et al., 2008). Johnson et al. (2018) provide a good overview of ML methods for expression data analysis.

For our analysis, we used data from the sRNA expression atlas (SEA; http://sea.ims.bio) (Rahman et al., 2017), a database containing well-structured manually curated ontology-based annotations of publicly available sRNA-Seq data. All data from the SEA were analyzed with the same workflow (OASIS; Rahman et al., 2018; https://oasis.dzne.de). We used 4243 annotated human sRNA-Seq samples from the SEA.

We applied the DL and RF classifiers for the considered augmentation problem and compared their results. The RF classsifier is an ensemble-based classifier, which outperforms other conventional classifiers for very high-dimensional data (Breiman, 2001). An RF classifier requires lesser training data than the DL classifier and allows the interpretation of features by generating variable importances. However, the RF classifier is sensitive to class imbalance (O'Brien and Ishwaran, 2019).

DL is able to analyze big data and is robust enough to treat large amounts of noisy training data (LeCun et al., 2015; Xiao et al., 2015). Its disadvantage is that it requires large amounts of training data (Li et al., 2019), is prone to overfit for small training sets, and is difficult to biologically interpret (feature importance) (Webb, 2018). In Kong and Yu (2018), the RF and DL approaches were used in two stages. For the first stage, the RF approach was used to extract the most important features, and then for the second stage, the DL approach was implemented for gene expression data classification based on the selected features. Many researchers are currently trying to explain DL models (Bach et al., 2015; Choi et al., 2016; Montavon et al., 2017). Some methods are model agnostic, which can explain the behavior of every “black box” or “gray box” model (Ribeiro et al., 2016; Lakkaraju et al., 2017; Molnar, 2019). Some methods are model specific, such as perturbation-based (Robnik-Sikonja and Bohanec, 2018) or backpropagation-based (Shrikumar et al., 2017) models. We have used DeepLIFT (Shrikumar et al., 2017) scores to explain DL models.

In this study, we present that DL algorithms outperform RF-based data augmentation for tissue, sex, and age annotations using sRNA expression profiles, if enough training data are available. More specifically, the DL method performs better than the RF method for cross-validation (CV) experiments as well as on “one data set out” experiments. We have demonstrated how backpropagation can provide a biological interpretation of relevant features for the DL classification.

## 2. Methods

### 2.1. Data and metadata acquisition

We augmented tissue, sex, and age based on human sRNA-seq expression profiles. We used sRNA-Seq data from the SEA (Rahman et al., 2017) that contains 4243 samples and annotations in 350 data sets. The relatively large number of high-quality samples allowed us to use DL for data augmentation. Each sample contained annotations, sRNA expression counts of ∼35,000 sRNAs, and expression information of potentially viral and bacterial transcripts (∼5600 contaminants), according to the output of the OASIS 2 sRNA analysis application (Rahman et al., 2018). Tissue prediction was based on sRNA expression profiles only, because the use of contamination profiles did not noticeably increase the accuracy of prediction; for age and sex prediction, contamination profiles were used. The number of data sets and samples is summarized in Table 1.

**Table 1.** Number of Samples and Data Sets Used to Augment Tissue, Age, and Sex

Metadata field	No. of data sets	No. of samples
Tissue	128	2806
Tissue after filtering	105	2215
Sex	41	1591
Age	27	888

The samples comprised 42% males and 58% females.

To avoid small classes with specific tissues, we merged the available tissues using BRENDA tissue ontology (BTO) in the SEA (Table 2) according to Fiosina et al. (2019).

**Table 2.** Tissue and Cell Line Grouping According to Ontologies

Tissue group	Contained tissues
blood_group	Blood, blood plasma, blood serum, peripheral blood, umbilical cord blood, serum, buffy coat, immortal human B cell, liver, lymphoblastoid cell
brain_group	Brain, cingulate gyrus, motor cortex, prefrontal cortex, neocortex
epithelium_group	Skin, dermis, epidermis, breast, oral mucosa, larynx
gland_group	Prostate gland, testis, kidney, bladder, uterine endometrium, tonsil, lymph node
intestine_group	Intestine, colon, ileal mucosa

Age is a continuous variable and its exact prediction is a regression problem that might be highly inaccurate when solely based on sRNA expression information. To use the same methods as those used for the prediction of other annotation fields, we grouped ages into *k* intervals, *k* = 2, 3, 4. Table 3 summarizes the intervals used for age prediction.

**Table 3.** Age Intervals Used in Prediction

Two intervals	Three intervals	Four intervals
[0;65], (65;110]	[0;45], (45;70], (70, 110]	[0;30], (30;60], (60, 80], (80, 110]

### 2.2. Data scaling and filtering

**FIG. 1.**
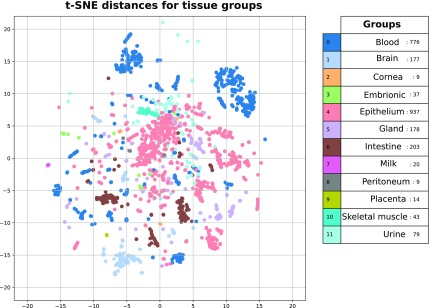
t-SNE plot for available tissue types. t-SNE.

Data scaling and filtering are described in detail in Fiosina et al. (2019). In brief, the counts were normalized using reads per million (RPM), each factor was normalized using a MinMax Scaler, the factors containing >30% zeros were removed (leaving ∼2500 sRNAs and 2000 contaminants). Small groups were also removed, leaving 105 data sets for the analysis (Table 1). We observed 23% cell lines and 77% tissue samples in our data. Figure 1 illustrates the T-distributed stochastic neighbor embedding (t-SNE) plot for the tissue groups after the sample filtering.

### 2.3. Models

#### 2.3.1. DL model

We used a fully connected neuronal network (NN) architecture. It comprised one input layer with the number of inputs equal to the number of variables after the initial filtering. The NN contained three hidden layers with 1000, 250, and 250 neurons and dropout rates of 0.5, 0.4, and 0.4, respectively (achieved by a grid search). The number of neurons in the output layer was equal to the number of predicted classes. The output data (the annotation classes) were encoded with integers (0, 1, 2, …, *num*_*of*_*classes*) and transformed to categorical variables consisting of 0's and 1's. We used the ReLU function to activate the input and the hidden layers, and the softmax function for the output layer, for a multiclass classification. As a loss function, either a binary or categorical cross-entropy was used. For the NN training, Adam optimizer was used. The NN was trained for 50 epochs with a batch size of 30.

#### 2.3.2. RF model

We used a two-stage classification strategy. First, we used all features (remained after filtering) for the classification and feature importance calculation. We used the top-1000 features rated by their Gini index for the second stage classification with an increased number of trees (500 instead of 100). The mtry parameter was equal to the square root of the number of features. Given that the performance of RF method can be strongly affected by class imbalance, we downsampled large classes to the size of the smaller classes.

#### 2.3.3. Validation

We implemented two types of CV to investigate the accuracy of the data augmentation. First, we used the average accuracy of fivefold CV. In this scenario, the training cells and test samples were randomly selected a priori, so that in most cases, samples from each data set (experiment) could be included in the training and test sets.

Then, we performed “one-data set-out” classifications, where specific data sets were removed from the training set and incorporated into the test set after ensuring that the respective tissues still remained in the training data set.

Throughout this article, we refer to the fivefold CV as “cross validation” and the validation for unseen data sets as “one data set out.”

#### 2.3.4. Deep lift

To biologically trace the decisions of the DL model to the input features, we used DeepLIFT scores. DeepLIFT (Shrikumar et al., 2017) is an approach to assign importance scores, which demonstrate how important the value of each particular input is for each particular output. The scores are assigned according to the difference between a given input and some reference (neutral) input. The DeepLIFT method overperforms other scoring methods (Shrikumar et al., 2017); thus, it was selected for our analysis. The DeepLIFT method calculates scores by backpropagating the contributions of all neurons in the network to every feature of the input. Consequently, for each sample *i*, each input neuron *j*, and each output neuron *k*, a score *C_i_*_, *j*, *k*_ is calculated, which represents an importance of an input *j* for an output *k* in the *i*-th input sample.

We have provided a three-step explanation of our augmentation models.

First, we used a heatmap to visualize the DeepLIFT scores of an individual sample. This enabled us to understand which sRNAs are important for a particular prediction.

Second, we analyzed important sRNAs for each class *k*. We selected samples, which belonged to the class *k*: *y_i_* = *k* and calculated the average difference scores for the correct class and other classes:


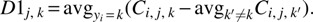


Then, we selected the top *N* sRNAs *j* according to *D*1_*j*, *k*_ for each class *k*.

Finally, we investigated the number of sRNAs to be removed (to set their expression to 0), to change the classification results. For each sample *i* of class *y_i_* = *k* and each class *k^′^* ≠ *k*, we calculated the score differences


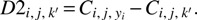


We ordered the differences *D*2(*i*, *j*, *k^′^*) and set the expression of sRNAs *j* 0. We stop the process when the classification changes from *k* to *k^′^* (similarity analysis) or to any other class *k*^″^≠*k* (stability analysis). The corresponding average number of steps was applied to a matrix, which demonstrated “stability” of class (or “class similarity”). As a reference input, we used a vector of 0's.

#### 2.3.5. Software libraries

All the scripts for DL classification are developed in R based on the “keras” library. The RF models are also implemented in R, using the “randomForest” library. For quality metrics, we used R “caret” package. We used the Python 3.5 “sklearn.manifold” t-SNE library to build the t-SNE plots. DeepLIFT was implemented using the “deeplift” Python library version 0.6.9.0.

## 3. Results

### 3.1. Tissue prediction

#### 3.1.1. Tissue group prediction

We aimed to predict the tissue class, that is, grouped tissue (2). Although grouping leads to a smaller number of classes, it increases the samples per class. This should reduce the problems due to class imbalances and the overfitting of very small training classes. The prediction was based on sRNA expression profiles only.

##### 3.1.1.1. CV experiments

**FIG. 2.**
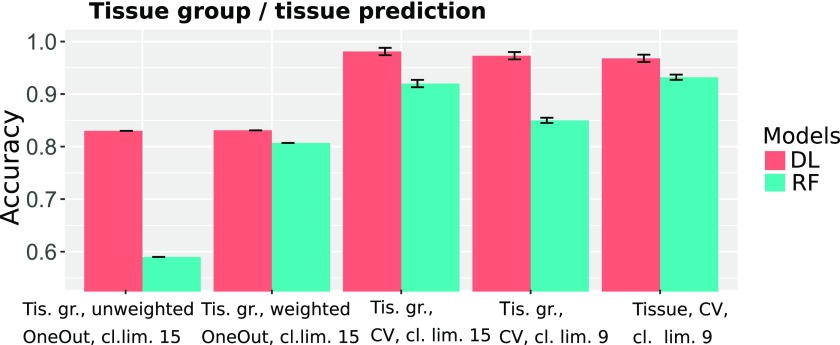
CV and “one data set out” accuracy of tissues and tissue groups. CV, cross-validation.

**FIG. 3.**
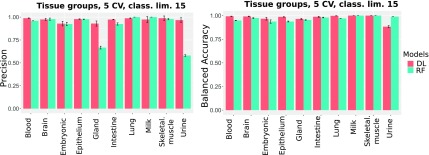
CV precision and accuracy for classes with minimum 15 samples.

**FIG. 4.**
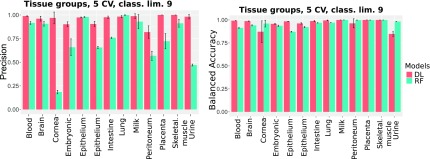
CV precision and accuracy for classes with minimum 9 samples.

To compare the performance of the DL and the RF models for data sets with a different degree of imbalance, we excluded classes for which <9 or 15 samples were available. Figure 2 shows that the RF model is less accurate, particularly for the threshold of 9 (DL: 97%, RF: 85%). For the threshold of 15, the accuracy increases; however, it is still significantly inferior to that of the DL models (DL: 98%, RF: 92%). We surmised that the better performance of the DL model, together with the fact that the accuracy is only slightly affected by the minimum class size, can be attributed to its resilience to class imbalances (Figs. 3 and 4).

##### 3.1.1.2. “One data set out” experiments

**FIG. 5.**
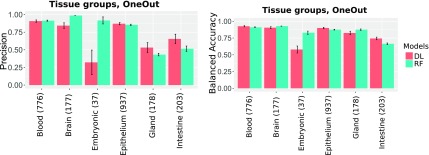
“One data set out” tissue group prediction precision (left) and accuracy (right).

As detailed in the Section 2, the aggregation of samples revealed six tissues with more than one data set per tissue (Fiosina et al., 2019, Sample Filtering). For the “one data set out” classification, one data set was removed from the training set and was only used for testing the classification accuracy, as can be observed from the first two bargroups of Figure 2. This resembles a real augmentation scenario in which a data set with an unknown bias is augmented by the ML algorithm. Although the data sets in the training and testing sets were derived from the same tissue, they, most probably, possessed very distinct biases that could have originated from varying library preparation methods, the biological conditions of the samples, cell types, and diseases. The average accuracy of each group detection was 83.1% (DL) and 80.7% (RF) as shown in Figure 5.

### 3.2. sRNA-seq sex prediction

**FIG. 6.**
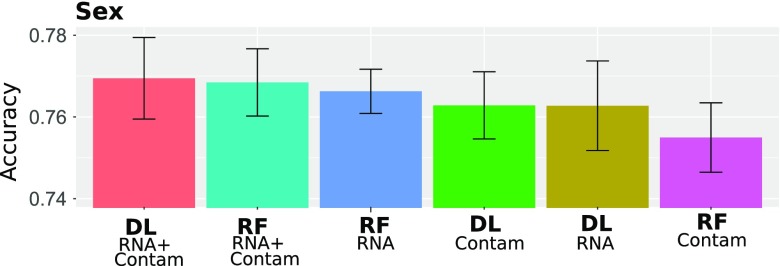
CV sex prediction accuracy with different models.

For the determination of sex, we opted for enlarging the data set with contamination expression counts. Effectively, we tried to predict sex with six different models: using sRNA expression counts, using contaminants, using both, each for the RF and the DL algorithm (Fig. 6). The best models were the DL and RF models based on both sRNAs and contaminations, with an accuracy of 77% and 76.9%, respectively. The other three models RF(RNA), DL(contaminations), and DL(sRNAs) demonstrated an accuracy of ∼76.2%. It was unexpected that the model based only on contaminations predicted the sex with an accuracy of ∼76% for both DL and RF models. Thus, for sex prediction, the DL model outperformed the RF model slightly.

### 3.3. sRNA-seq age prediction

**FIG. 7.**
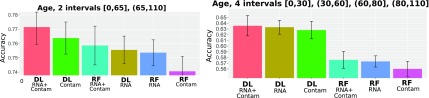
CV age prediction accuracy with different models.

For predicting the age, we used the contamination expression counts similar to sex prediction. We predicted the age categories for three different splits yielding two and four categories (Table 3). The results are presented in Figures 6 and 7.

A comparison of results using two and four categories demonstrates that accuracy decreases with increasing number of age categories for all models. In both cases, the DL models slightly outperform the RF model, in particular for a split into four intervals. Combined sRNA and contaminant data for a DL model yielded the highest accuracy, that is, 77.1% for a binary output and 63.5% for four intervals. Notably, DL using less data (sRNA or contaminant data only) presents an accuracy of 76.4% for binary output and 63.2% for four intervals. The RF models performed slightly worse on average with a maximum accuracy 75.8% for binary output and four intervals and 57.5% for four intervals.

### 3.4. Explanation of DL results

DL-based models are called “black boxes” because it is often unclear how the models arrive at their decisions. However, particularly in biological and medical settings, it is important to understand what enables algorithms to classify a sample, as the feature may be related to a cause as well as to a possible treatment. We investigated the explainability of automatic metadata augmentation with DL models, using backpropagation with the DeepLIFT method.

#### 3.4.1. Prediction explanation for individual samples

**FIG. 8.**
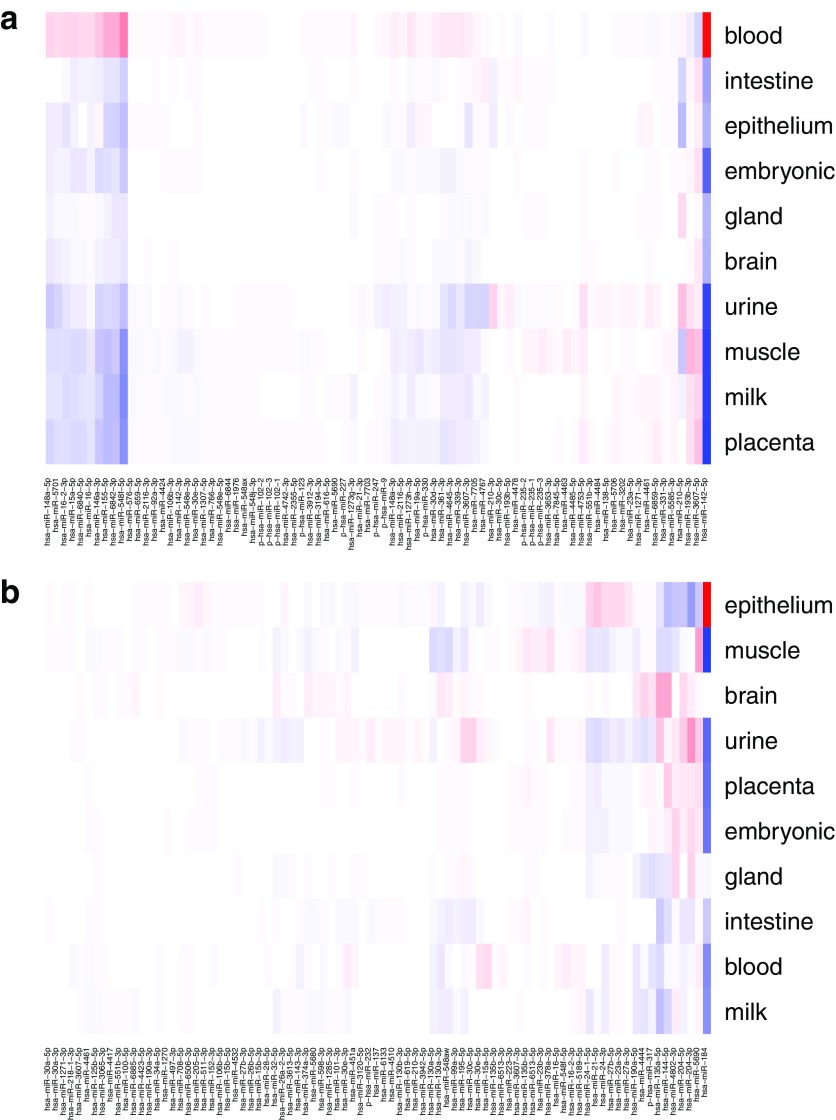
DeepLIFT scores for tissue group classification. On the left, we see some sRNAs clearly voting for the blood group (one sRNA on the right, and a cluster on the left). Particular sRNAs vote against the blood group (second and third on the left). Similarly, other tissues have specific sRNAs the score for or against the tissue. **(a)** Blood group sample **(b)** Brain group sample.

**FIG. 9.**
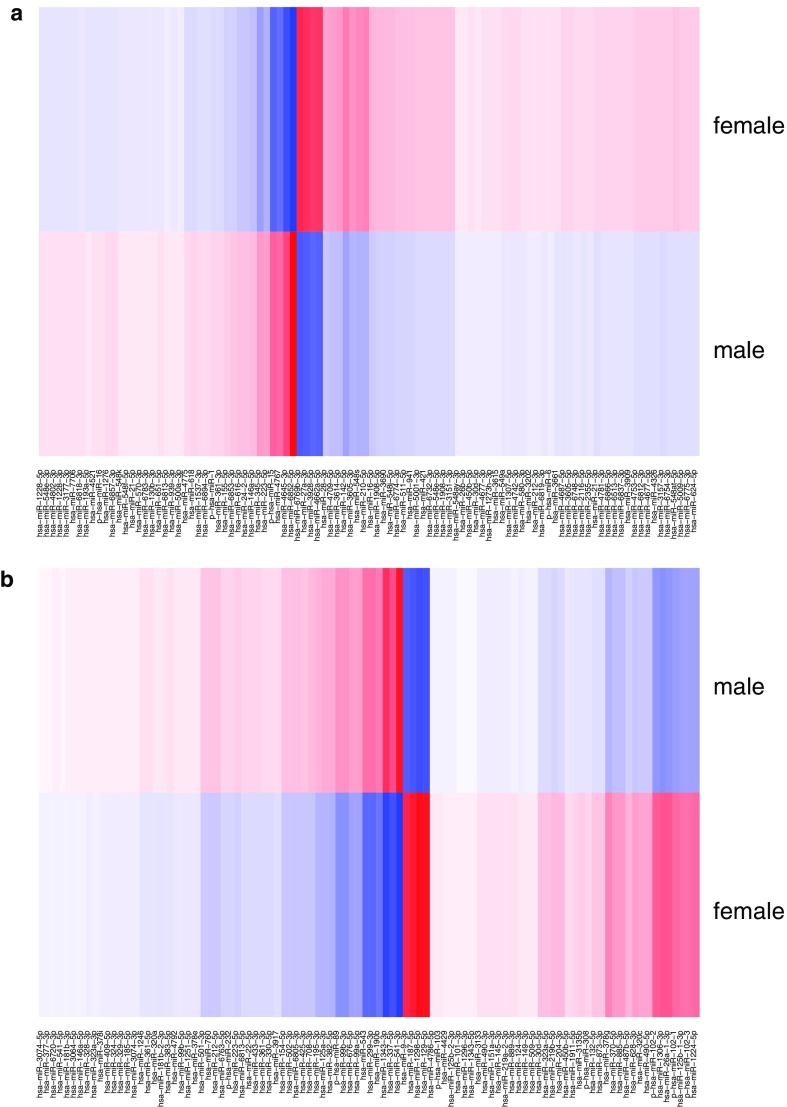
DeepLIFT scores for sex classification. For both samples, we see a number of sRNAs voting for and against each class. Both are classified as female. On the left, there is a majority of sRNAs voting for female. **(a)** Female sample (correctly classified); **(b)** Female sample (incorrectly classified).

**FIG. 10.**
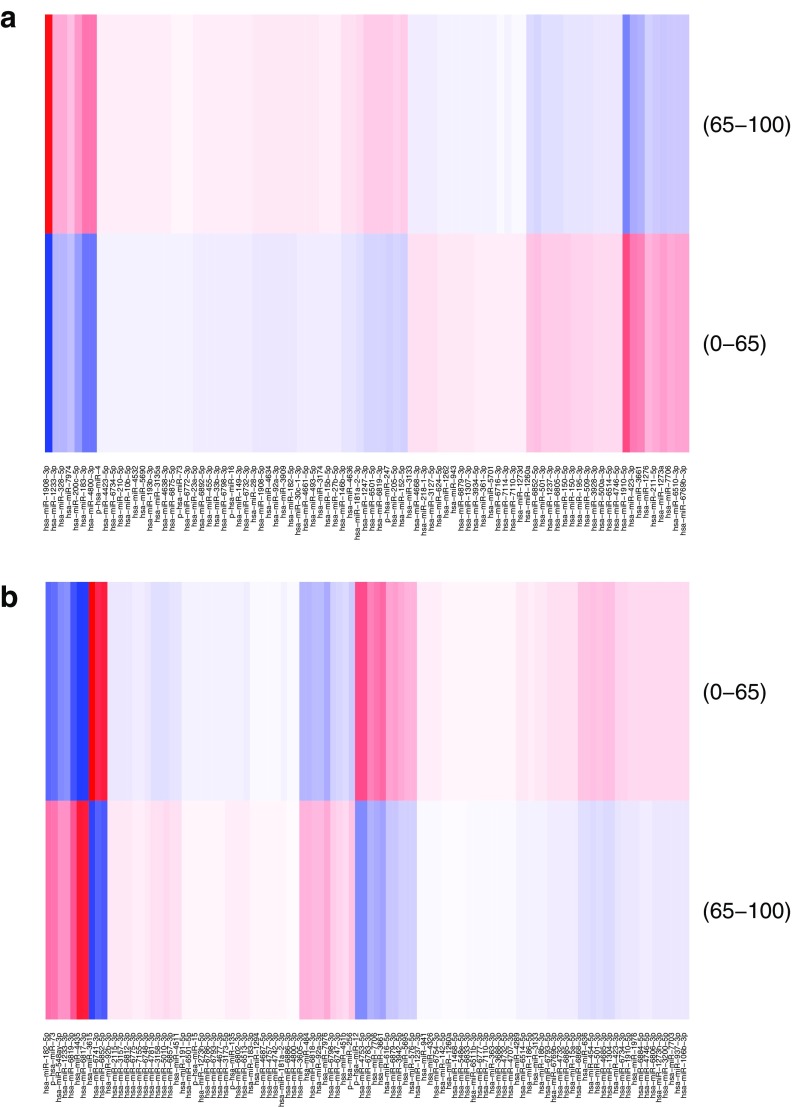
DeepLIFT scores for age classification. For both samples, we see a number of sRNAs voting for and against each class. However, the left sample has more sRNAs that vote for the old class and the right sample has more sRNAs that strongly vote for the young class. **(a)** Old sample (correctly classified); **(b)** Young sample (correctly classified).

To visualize the backpropagation results, we used heatmaps that represented the scores for each individual class (Figs. 8–10).

The figures demonstrate that the visual representations of DeepLIFT scores may explain the factors important for a particular output of the NN. This shows that deep neural networks can indeed offer explainable and interpretable results.

#### 3.4.2. Average scores for sample prediction and enrichment

**FIG. 11.**
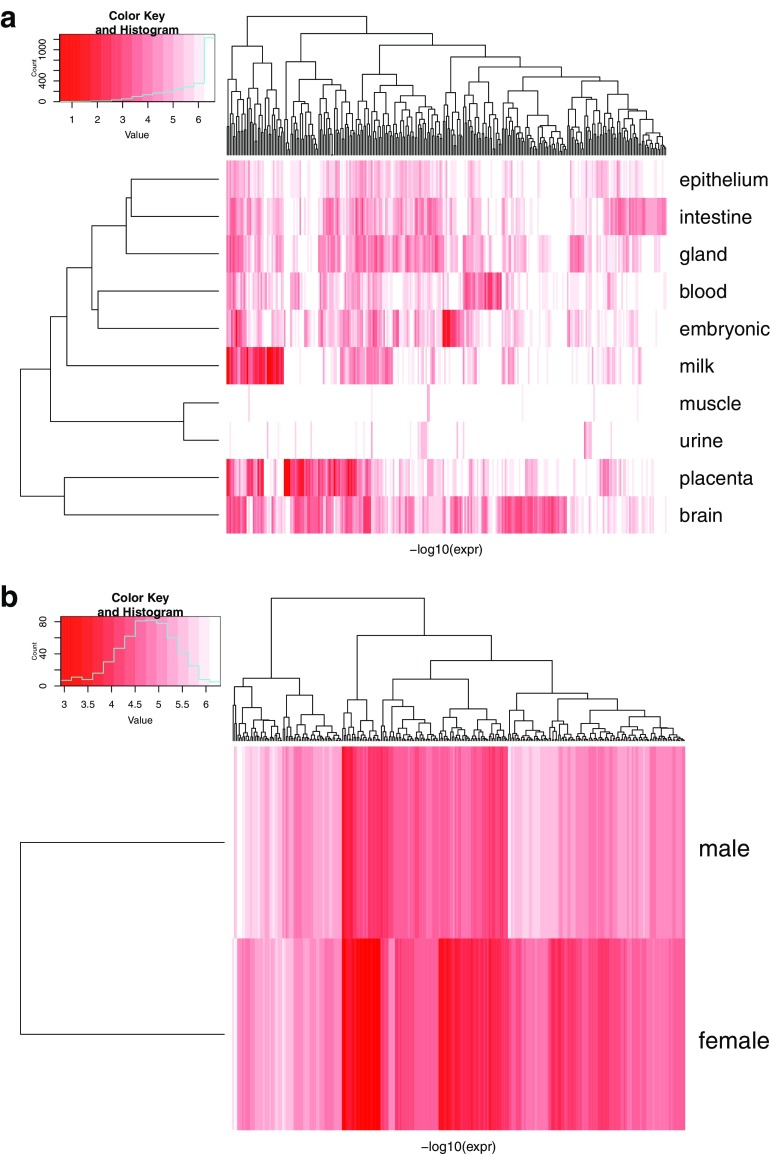
Expression of sRNAs with top 300 DeepLIFT scores. **(a)** Tissue group prediction; **(b)** sex prediction.

The most important sRNAs for a class cannot, however, be determined on a per-sample basis as individual samples show rather large variations. Thus, we computed *D*1_*j*, *k*_ for each tissue *k* as outlined in Section 2.3.4. Using these scores, we selected the top *N* = 300 sRNAs *j* and calculated the average expression for each class (Fig. 11).

We observed that factors with big average DeepLIFT scores do not show a clear separation by expression levels. We still see some clusters of sRNAs, which are characteristic for the groups. These observations may be explained by nonlinear class separation of the DL, which is not reflected just by average expression per class.

**FIG. 12.**
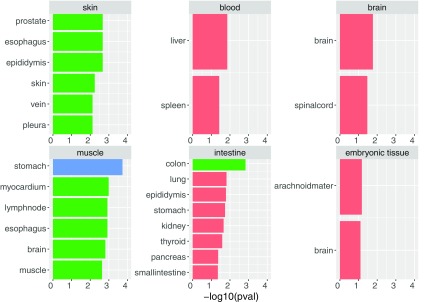
Tissue enrichment. We see enrichment in six categories. For most of them, we see enrichment of tissue-relevant terms. For example, for epithelium we see skin, vein, and pleura categories, for blood—liver and spleen, which are blood producing organs; for brain—brain and spinal cord, which are central nervous system relevant.

To make sure that the results contain biologically relevant sRNAs, we investigated the enrichment of biological categories based on important sRNAs. We used the model based on miRNA only, as the enrichment information is available mostly for miRNAs (Fig. 12 and Table 4).

**Table 4.** Enriched miRNAs for Tissue Prediction

Category	Enriched miRNAs
Skin	hsa-miR-205-5p; hsa-miR-205-3p; hsa-miR-193a-5p; hsa-miR-23a-3p; hsa-miR-21-5p; hsa-miR-3195; hsa-miR-27a-3p; hsa-miR-224-5p; hsa-miR-98-5p; hsa-miR-944
Blood	hsa-miR-99a-5p; hsa-miR-142-5p; hsa-miR-4732-3p; hsa-miR-486-5p; hsa-miR-15a-5p; hsa-miR-1976; hsa-miR-16-5p; hsa-miR-16-2-3p; hsa-miR-129-5p; hsa-miR-1224-5p
Brain	hsa-miR-153-3p; hsa-miR-138-5p; hsa-miR-100-5p; hsa-miR-9-5p; hsa-miR-874-3p; hsa-miR-124-3p; hsa-miR-125b-5p; hsa-miR-181c-3p; hsa-miR-654-3p; hsa-miR-598-3p
Muscle	hsa-miR-378a-5p; hsa-miR-133a-3p; hsa-miR-193b-3p; hsa-miR-4463; hsa-miR-6723-5p; hsa-miR-4644; hsa-miR-1271-5p; hsa-miR-378a-3p; hsa-miR-4485-3p; hsa-miR-193b-5p
Intestine	hsa-miR-215-5p; hsa-miR-194-3p; hsa-miR-194-5p; hsa-miR-192-3p; hsa-miR-192-5p; hsa-miR-200b-3p; hsa-miR-200b-5p; hsa-miR-19b-3p; hsa-miR-31-5p; hsa-miR-200c-3p
Embryonic tissue	hsa-miR-92b-3p; hsa-miR-18b-3p; hsa-miR-363-3p; hsa-miR-421; hsa-miR-3195; hsa-miR-335-3p; hsa-miR-887-3p; hsa-miR-3648; hsa-miR-4417; hsa-miR-130b-3p

Our enrichment analysis clearly shows an overrepresentation of biologically meaningful sRNAs for a given target tissue, demonstrating that DeepLIFT scores allow the extraction of important tissue-specific sRNAs. We conclude that DeepLIFT is a viable method to explain DL decisions for genomic data.

#### 3.4.3. Stability of solution

**FIG. 13.**
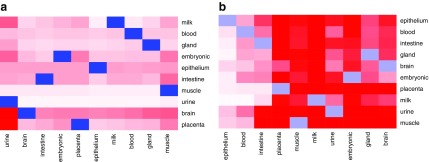
Stability and similarity of classes for tissue group classification. Intensity of red color shows number of steps to change class. Some classes are stable, such as the brain, intestine, and epithelium. Other classes such as the urine, skeletal muscle, or gland are quite unstable and are easily transformed to the epithelium or blood groups. **(a)** Class stability; **(b)** class similarity.

Furthermore, we wanted to assess whether DeepLIFT scores could provide insights into the stability of DL models. We ordered the differences *D*2_*i*, *j*, *k′*_ and calculated the number of steps to change the predicted class according to Section 2.3.4 (Fig. 13 and Table 5). Note that some classes are stable, other classes are quite unstable. These results most probably reflect the specificity and quantity of group-specific feature expression levels.

**Table 5.** Average Number of sRNAs to Change for Changing Sex and Age Prediction

Classification model	Class 1	class 2	No. of steps class1 into class2	No. of steps class2 into Class1
Sex prediction	Female	Male	15.9	13.7
Age prediction	[0–65]	[65–110]	8.6	15.0

## 4. Conclusion and Future Work

Depending on the outcome variable (e.g., tissue, sex, and age), automatic metadata augmentation can be a good option to annotate the missing metadata using sRNA expression levels. The DL-based classification accuracy of tissue and sex predictions reaches 98% and 77%, respectively, the classification of age groups (or the regression of age) seems to demonstrate an inferior performance. In general, metadata augmentation, as undertaken in this study, is dependent on the occurrence of the tissue of interest, or a similar tissue, in the training data set of the classifier. Another general problem is that of class imbalances and very rare classes. In this study, we have used an ontology-based grouping of rare classes to higher ontological nodes to increase the number of samples for a given class. In future work, we plan to use a hierarchical classification, from general to specific tissue classes, to investigate the classification performance across the ontological hierarchy. We have also demonstrated that, in general, the inclusion of contamination profiles in classification models improves the accuracy for sex and age.

sRNA expression profiles seem to be suitable for the augmentation of tissue information. A CV-based tissue group classification achieves an accuracy >98%. In the “one data set out” scenario, with a specific data set with a specific bias missing from the training data, samples from the unseen data set are classified with an accuracy of ∼80%.

For sex classification, the DL model achieved an accuracy of ∼77%, which may not be sufficient for accurate sex classification. This relatively low accuracy indicates that there may be no sex-specific expressed sRNAs for the X- or Y-chromosomes. Similarly, we obtained an accuracy of 77% for predicting whether a person is younger or older than 65 years. For a split into four intervals, accuracy decreased to 64%, indicating that the sRNA transcriptome does not consistently change with age.

Lastly, we demonstrated that DL models can be explained both for individual samples and on average. For this purpose, the DeepLIFT scores demonstrated very promising results.
